# *XC_0531* encodes a c-type cytochrome biogenesis protein and is required for pathogenesis in *Xanthomonas campestris* pv. *campestris*

**DOI:** 10.1186/s12866-017-1056-9

**Published:** 2017-06-27

**Authors:** Lei Chen, Mingpeng Wang, Li Huang, Zhaojie Zhang, Fanghua Liu, Guangtao Lu

**Affiliations:** 1 State Key Laboratory for Conservation and State Key Laboratory for Conservation and Utilization of Subtropical Agro-Bioresources, The Key Laboratory ofMinistry of Education for Microbial and Plant Genetic Engineering, Guangxi University, 100 Daxue Road, Nanning, Guangxi 530004 China; 2 Key Laboratory of Coastal Biology and Biological Resources Utilization, Yantai Institute of Coastal Zone Research, Yantai, China; 3 Department of Zoology and Physiology, University of Wyoming, Laramie, WY USA

**Keywords:** Cytochrome c, Eps, *Xanthomonas campestris*, Pathogenesis

## Abstract

**Background:**

The phytopathogenic *Xanthomonas campestris* pv.*campestris* is a gram-negative bacterium and the causal agent of black-rot disease of cruciferous crops. Many gram-negative bacteria possess a family of proteins, called Dsbs, which are involved in disulfide bond formation in certain periplasmic proteins. In our preliminary screening of the virulence to the plants we identified that gene *XC_0531* which annotated gene *dsbD* of *Xanthomonas campestris *pv. *campestris* (*Xcc*) is related to the virulence to the host plants.

**Results:**

Here, we found *XC_0531* encoded a DsbD like protein. Its deletion is sensitive to DTT and copper, decreased accumulation of free thiols in periplasm. Its deletion also affected heme synthesis, position of Soret band and the production of peak c550. This suggests that *XC_053*1 is related to c-type cytochromes biogenesis. *XC_0531* mutation decreased the utilization of different carbon sources (such as galactose, xylose, maltose, saccharose and glucose), reduced extracellular polysaccharide (EPS) production, decreased extracellular enzyme activities (protease, cellulose and amylase), slowed down growth rate of *Xcc* and weakened virulence to the plants. These results suggest that these phenotypes caused by *XC_0531* mutation is possibly due to deficient biosynthesis of c-type cytochromes in respiration chain and the formation of disulfide bonds. Our work confirmed the function of *XC_0531* and provide theory basis for scientists working on molecular mechanisms of cytochrome c biogenesis, pathogenesis of *Xcc*, development of EPS commercial values and protecting plant from black rot.

**Conclusion:**

We confirmed the function of gene *XC_0531*, which encodes a DsbD like protein, a protein correlated with c-type cytochrome biogenesis. This gene is related to the virulence to plants by affecting funtion of cytochromes c and probably disulfide bonds modification of proteins in type II secretion system (T2SS).

**Electronic supplementary material:**

The online version of this article (doi:10.1186/s12866-017-1056-9) contains supplementary material, which is available to authorized users.

## Background

The phytopathogenic *Xanthomonas campestris* pv. *campestris* is a gram-negative bacterium and the causal agent of black-rot disease of cruciferous crops. Many gram-negative bacteria possess a family of proteins, called Dsbs, which are involved in disulfide bond formation in certain periplasmic proteins [1]. These disulfide bonds, which are structurally modified via disulfide bond formation in the periplasm, are critical for the maturation of virulence factors. The Dsb proteins also have thiol:disulfide oxido-reductase activities [2–7]. DsbA is a strong oxidant with a weak isomerase activity in the periplasmic space for disulfide bond formation [2, 8–10]. DsbB together with DsbA catalyze the formation of cysteine thiols [2]. DsbC has a disulfide isomerase activity catalyzing disulfide formation [10, 11]. DsbD acts as a reductase in the periplasmic space [12]. DsbD also participates in the biosynthesis of cytochromes c [1].

C-type cytochromes act as a partner in the respiration chains and play an important role in the metabolism of bacteria [13] and fungi [14, 15]. C-type cytochromes are distinguished from cytochromes of the other classes by covalent attachment of the heme group to the conserved CXXCH motif of the apocytochromes. The biogenesis of c-type cytochromes has two systems class I and class II. Most gram-negative bacteria harbor the class I system including at least 11 proteins (Dsb ABD and Ccm ABCDEFGH). The heme group attachment to the apoprotein takes place in the oxidative environment of the periplasm [2]. Thus, c-type cytochromes apoproteins need to be reduced prior to heme binding by CcmH, CcmG and DsbD in cytochrome c maturation in *Escherichia coli* [2, 16].

Our previous studies have shown that DsbB proteins play important roles in pathogenesis of *Xcc* [17] and in a preliminary screening for pathogenic genes we found that gene *dsbD* is needed for the virulence of *Xcc* to plants. Here, we confirmed the function of gene *XC_0531*, which encodes DsbD, a protein correlated with c-type cytochrome biogenesis and is related to the virulence to plants.

## Materials and methods

### Bacterial strains, plasmids and growth conditions

The bacterial strains and plasmids used in this study are listed in Table 1. *E. coli* strains were grown in LB medium at 37 °C. *Xcc* strains were grown at 28 °C in NYG medium (5 g of peptone, 3 g of yeast extract and 20 g of glycerol per liter), NYGA medium (NYG medium with 1.5% agar), NY medium (NYG medium without glycerol) or non-carbohydrate minimal medium MMX [18]. Antibiotics were added at the following concentrations as required: kanamycin (Kan), 25 μg ml^−1^; rifampicin (Rif), 50 μg ml^−1^; ampicillin, 100 μg ml^−1^; spectinomycin, 50 μg ml^−1^; tetracycline (Tet), 5 μg ml^−1^ for *Xcc* and 15 μg ml^−1^ for *E. coli*.

**Table 1:** Strains and plasmids used in this study

Strains and Plasmids	Genotype/Properties	Reference/Resource
*Escherichia coli*		
JM109	*RecA*1, *endA*1, *gyrA*96, *thi*, *supE*44, *relA*1*△* (*lac*-*proAB*)/F′ [*traD*36, *lacI* ^q^, *lacZ△*M15]	Our lab’s collection
ED8767	RecA, met,containing pRK2073,Spc^r^	Our lab’s collection
M15	*Kmlac ara gal mtl recA1 uvr1* [pREP4 lacI Kanr]	Our lab’s collection
*Xanthomonos campestris* pv. *compestris*		
8004	Wild type; Rif ^r^	Our lab’s collection
015F07	As 8004,but *XC_357*9::Tn5*gus*A5;Rif^r^, Km^r^	This work
045F12	As 8004, but *XC_3579*::Tn5*gus*A5;Rif^r^, Km^r^	This work
142E11	As 8004, but *XC_3579*::Tn5*gus*A5;Rif^r^, Km^r^	This work
0531pk	As 8004, but *XC_0531*::pK18mob; Rif^r^, Km^r^	This work
C0531	0531pk harboring pLA0531; Rif^r^, Km^r^, Tet^r^	This work
Plasmids		
pK18*mob*	pUC18 derivative, *lacZα*, Kan^r^, mob site, suicide plasmid in *X. campestris* pv. *campestris*.	Our lab’s collection
pRK2073	Helper plasmid,Tra^+^, Mob^+^, ColE1, Spc^r^.	Our lab’s collection
pET30a^+^	T7 promoter, Km^r^	Our lab’s collection
pLC0531	pLAFR3 containing the whole XC_0531 gene;Tc^r^	This work
pLAFR3	Broad host range cloning vector, Tc^r^	Our lab’s collection

### DNA manipulations

Methods as described previously [19] were used for preparation of plasmid and chromosomal DNAs, restriction digestion, DNA ligation, agarose-gel electrophoresis and DNA transformation of *E. coli*. Conjugation between *Xcc* and *E. coli* strains was performed as described previously [20]. Restriction endonucleases, T4 DNA ligase and Pfu polymerase were purchased from Promega.

### Insertional mutant construction and complementation

An insertional mutant of the ORF *XC_0531* was constructed using the suicide plasmid pK18*mob* [21, 22] as described previously [23]. A 539 bp internal fragment of the *XC_0531* ORF sequence was amplified by PCR using the total DNA of wild-type *Xcc* strain 8004 as a template. The following pairs of oligo nucleotides were used as primers (Additional file 1: Table S1). Primers were modified to provide BamHI-HindIII ends. The amplified DNA fragments were cloned into pK18*mob* in the same orientation as the lacZ promoter. The resulting recombinant plasmid was introduced from *E. coli* strain JM109 [24] into *Xcc* wild-type strain 8004 by triparental conjugation, using pRK2073 as the helper plasmid [25]. The mutant was confirmed by PCR using the primers P18conF and 0531F (Additional file 1: Table S1). The obtained mutant strain was named 0531pk (Table 1). For complementation of mutant 0531pk, a 2786 bp DNA fragment containing the *XC_0531* ORF was amplified using the following pair of oligonucleotides as primers: C0531-F and C0531-R (Additional file 1: Table S1). Primers were modified to give BamHI- or HindIII-compatible ends. The amplified DNA fragment was cloned into plasmid pLAFR3 [26]. The obtained recombinant plasmid pLC0531 was transferred into the mutant strain 0531pk by triparental conjugation, resulting in the complemented strain named C0531 (Table 1). The C0531 strain was confirmed by PCR using the primers P18conF and C0531F (Additional file 1: Table S1).

### Virulence assay

The virulence of *Xcc* to the host plant Chinese radish (*Raphanus sativus*) was tested by the leaf-clipping method [27]. Lesion length was measured 10 days after inoculation.

### Sequence analyses

The amino acid sequences of DsbD and DsbC proteins of *Xcc*, *Escherichia coli* str. K-12, *Shewanella oneidensis* MR-1 and *Pseudomonas aeruginosa* PAO1 were obtained from the database of the National Center for Biotechnology Information (NCBI). Prediction of transmembrane helices was performed with TMHMM Server v.2.0 software [28].

### Heme staining

SDS-PAGE was carried out without the addition of DTT. Coomassie brilliant blue staining was carried out as described previously [29]. TMBZ was dissolved in methanol to a final concentration of 6.3 mM. The gel was covered with a solution of 3 parts TMBZ and 7 parts 0.5 M sodium acetate and incubated in the dark for 20 min. H_2_O_2_ was added to the final concentration of 30 mM for the visible protein gel bands, as describes [30].

### Analysis of c-type cytochromes

*Xcc* strains were cultured for 14 ~ 16 h to the exponential phase of growth in the NYG and then cellular membranes were prepared from cultures grown for 24 h on minimal medium (MMX) for *Xanthomonas campestris* with 1% mannitol. The cells were harvested by centrifugation, washed in 0.1 M phosphate buffer (pH 7.4), and resuspended in 0.1 M phosphate buffer (pH 7.4) with 30% (vol/vol) glycerol.

Total soluble protein fractions were prepared by sonication of washed cells in 0.1 M phosphate buffer (pH 7.4). Sonicates were centrifuged to remove unbroken cells at 10,000×g for 15 min at 4 °C. The soluble part and membrane fractions were separated by ultracentrifugation at 100,000×g for 2 h. The soluble proteins were resuspended in 10 mM Tris-HCl (pH 7.4) and membrane fractions were resuspended in 16 mM Tris-HCl (pH 7.4).

Cytochrome spectra were recorded at room temperature using a Beckman DU730 spectrophotometer (Beckman Coulter Inc.). The samples were diluted by PBS and reduced with a few granules of sodium dithionite or oxidized with ammonium persulfate. Reduced minus oxidized spectra were obtained by recording differences between the spectra of the dithionite-reduced sample and the ammonium persulfate-oxidized sample. Protein concentrations were measured with a protein assay kit (Pierce), using BSA as the standard. Proteins were separated by electrophoresis through 15% SDS-polyacrylamide gels by mixing with dithiothreitol (DTT)-free 5 × SDS loading buffer. Samples prepared with buffer containing DTT followed by heating resulted in a loss of staining of c-type cytochromes. Highest levels of heme stain signal were obtained using DTT-free buffer and no heat.Proteins containing covalently bond heme iron were visualized using 3,3′,5,5′- tetramethyl benzidine (TMBZ), as described [30].

### Ellman’s assay

Periplasmic protein samples from bacterial cells were prepared using the chloroform method with minor modification [17]. Cells were grown in NYG at 28 °C to the optical density of OD_600_ = 0.5, 500 ul cultures were centrifuged at 1000 rpm for 10 min to remove the supernatant. The strains were treated with chloroform for 15 min, and then 200 ul of 0.8 mM DTNB (5, 5′-dithiobis-[2-nitrobenzoic acid]) was added into the samples for 5 min. The supernatant containing periplasmic protein was obtained by centrifugation at 6000 rpm for 20 min. The absorbance was determined at 412 nm. The assays were carried out in three independent experiments.

### DTT sensitivity tests

For the DTT sensitivity test, 2.5 μl of overnight cultures of each strain OD_600_ = 1.0 were spotted onto NYG plates supplemented with 4 mM DTT and incubated at 28 °C for 48 h. At least three plates were inoculated for each strain and each experiment was repeated three times.

### EPS assay

To estimate EPS production, strains were cultured in 100 ml NY medium supplemented with 2% (*w*/*v*) of various sugars at 28°C with shaking at 200 rpm for 3 days. EPS was precipitated from the culture supernatant with ethanol, dried and weighed as described [31, 32].

### Motility assay

To test the swarming motility, 3 μl of overnight culture (OD_600_ of 1.0) of each *Xcc* strain was inoculated onto NY plates containing 2% glucose and 0.6% agar, and then incubated at 28 °C for 4 days. The diameters of the area occupied by the bacterial cells were measured and these values were used to indicate the motility of the *Xcc* strains [33]. The experiment was repeated at least three times.

### Extracellular enzyme activity analysis

To estimate the activity of the extracellular enzymes endoglucanase (cellulase), amylase and protease, *Xcc* strains were cultured in NYG medium for 12 h. For protease, 3 μl of overnight cultures (OD_600_ of 1.0) was spotted onto NYGA plates containing 1% skim milk, after incubation at 28 °C for 24 h, plates were photographed. For cellulose, 3 μl of overnight culture was spotted onto NYGA plates containing 0.5% carboxymethyl cellulose after incubation at 28 °C for 48 h, plates were stained with I2/KI (0.08 M I_2_, 3.2 M KI) and washed by 70% ethyl alcohol. For amylase, 3 μl of overnight cultures was spotted onto NYGA plates containing 0.1% starch incubation at 28 °C for 24 h. The plates were stained with 0.1% Congo Red and then washed 2 times. At last plates were destained using 1 M NaCl solution. For extracellular protease activity, the method was as described previously [34]. For cellulase (endoglucanase), 10 μl of enzyme-containing extracts was added to 200 μl of indicator buffer containing 1% (wt/vol) carboxymethyl cellulose (CMC, Sangon, Shanghai, China) as the substrate. The reactions were carried out for 30 min at 28 °C. The released reducing sugars were measured as D-glucose equivalents, as described [17]. One unit (U) of the cellulase (endoglucanase) activity was defined as the amount of enzyme releasing 1 μM of reducing sugar per minute. Amylase activity quantification was conducted in the same way as for the cellulase (endoglucanase) measurement, except that the substrate was replaced by 1% (wt/vol) starch solution.

### Copper stress response analysis and RT-qPCR analysis

The overnight cultures of *Xcc* (OD_600_ of 1.0) were cultured in NYG medium with different concertration of CuSO_4_ (0.4 mM, 0.8 mM, 1.2 mM and 1.6 mM) for 2 days. The OD_600_ values were measured and recorded.

Real-time quantitative PCR (RT-qPCR) analysis was carried out and refered to our previous study [32]. Primers were list in Additional file 1: Table S1. All RT-qPCRs were performed in triplicate.

### Data analysis

One-way analysis of variance (ANOVA) was used to detect significant differences between the treatments. These analyses were carried out with SPSS 22.0 software.

## Results

### Disruption of *XC_0531* reduces virulence to the host plant

**Fig. 1:**
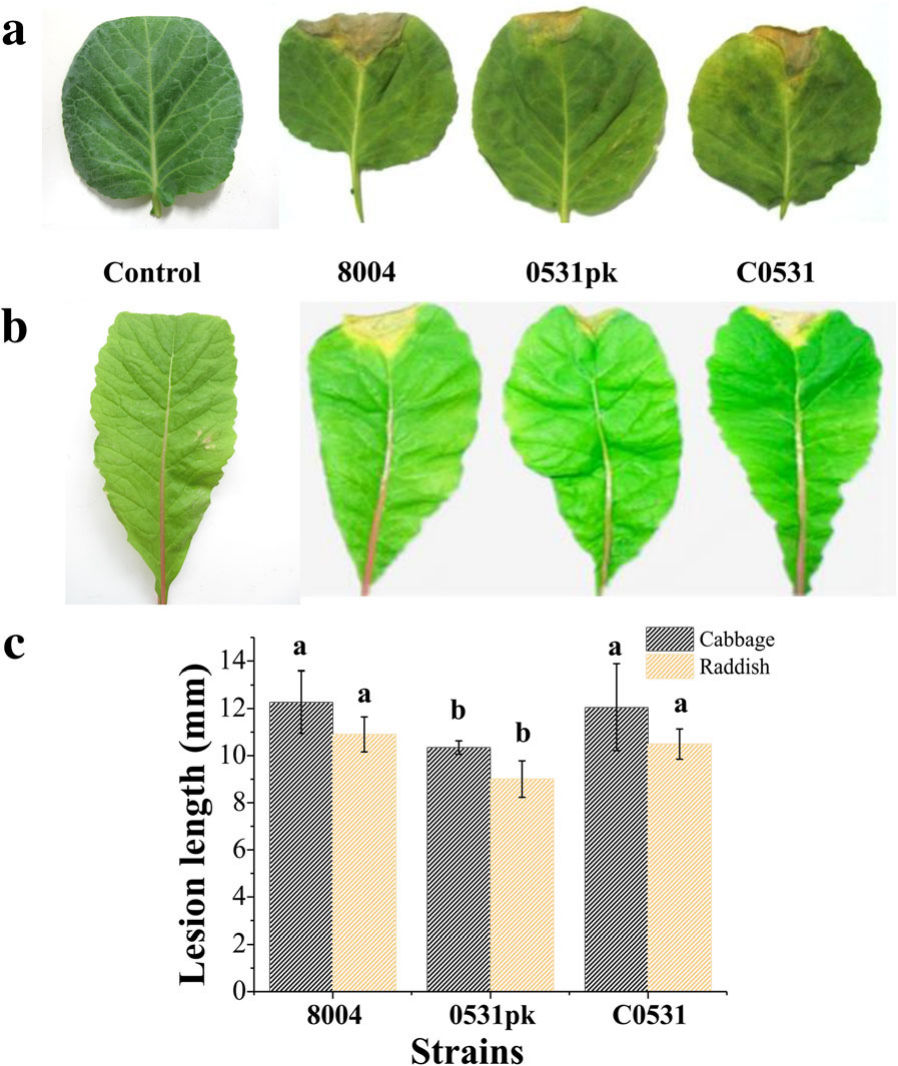
Pathogenic phenotype of the mutant in cabbage and radish. **a** and **b** Symptom production on leaves 10 days after inoculation by clipping with strains 8004, 0531pk and C0531. **c** Mean lesion lengths caused by different *Xcc* strains in cabbage and radish. Values are calculated from ~30 measurements. Different letters within a plant indicate significant differences at a level of *p* < 0.05 based on Duncan’s test by one way-ANOVA

In a preliminary screening, we found that gene *XC_0531* of *Xcc* is related to virulence to plants (unpublished data). To further confirm whether *XC_0531* is involved in the pathogenicity, the virulence of *Xcc* was tested on the host plants cabbage and radish by the leaf-clipping method [27]. As shown in Fig. 1, the wild type (strain 8004) produced a lesion length of 12.27 ± 1.33 mm in cabbage 10 days after inoculation. The lesion length was significantly reduced to 10.35 ± 0.28 mm in the mutant (0531pk) (*P* < 0.05). The complemented strain (C0531) induced lesion length similar to the wild type (12.05 ± 1.23 mm) in cabbage (Fig. 1a and c). Similar results were obtained in radish leaves (Fig. 1b and c). These results suggest that *XC_0531* plays an important role for *Xcc* virulence to plants.

### The ORF *XC_0531* of *Xcc* encodes a c-type cytochrome biogenesis protein

**Fig. 2:**
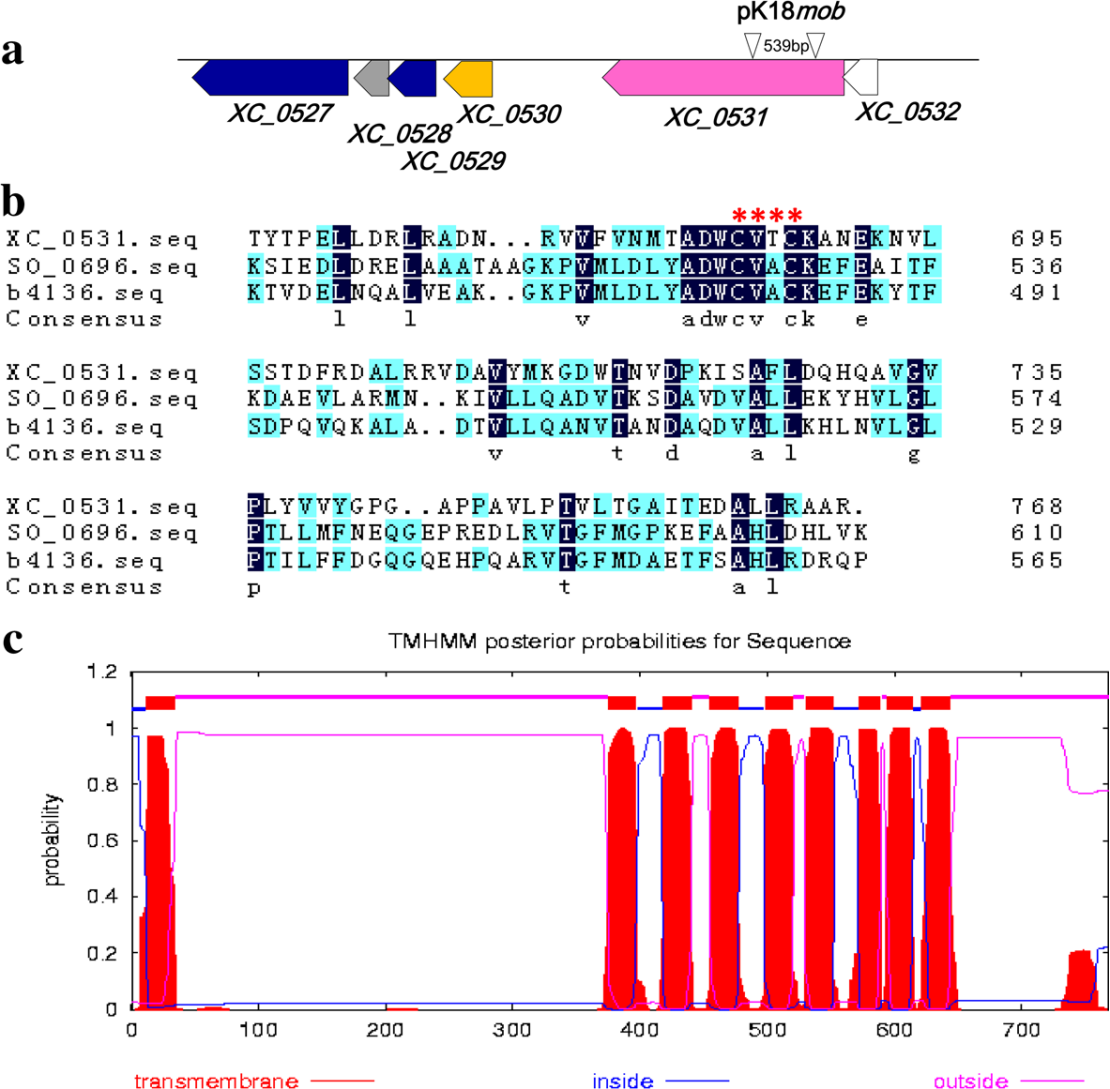
Sequence analyses of *XC_0531* of *Xanthomonas campestris* pv. *campestris*. **a** Physical and genetic map of the *Xanthomonas campestris* pv. *campestris dsbD* region. The pink arrowhead stands for the *dsbD* encoding open reading frame *XC_0531*. The small arrows represent the position of primers used to construct of the 0531pk. **b** Alignment of amino acid sequence of DsbD homologs from *Xanthomonas campestris* pv. *campestris* 8004, *Shewanella oneidensis* MR-1and *Escherichia coli* str. K-12. The gene *XC_0531* of *Xanthomonas campestris* pv. *campestris*, *SO_0696* of *Shewanella oneidensis* MR-1 and *b4136* of *Escherichia coli* str. K-12 were aligned. Navy blue boxes denote identical amino acid residues, whereas baby blue boxes are similar between the two sequences. The starred area denotes the typical redox active -Cys-X-X-Cys- motif of the disulfide bond formation protein family. **c** Prediction of transmembrane helices of *XC_0531* of *Xanthomonas campestris* pv. *campestris*. This was analyzed with TMHMM Server v.2.0 software. Red blocks stand for the transmembrane helices in cytoplasmic membrane while pink threads for periplasmic loops and blue for regions inside the cytoplasm

The protein sequence encoded by *XC_0531* of *Xcc* (strain 8004) (NCBI-Protein ID: AAY47612) was annotated as a c-type cytochrome biogenesis protein (http://xgb.leibniz-fli.de). It had 29% identity and 43% similarity to the thiol:disulfide interchange protein DsbD (NCBI-Protein ID: NP_418559, encoded by the gene b4136 in *Escherichia coli* str. K-12, and 28% identity and 43% similarity to DsbD of *Shewanella oneidensis* MR-1 (NCBI-Protein ID: NP_716329) encoded by gene *SO_0696*. The ORF *XC_0531* is located between 626,891 and 629,197 of the *Xcc* 8004 [35]. The ORFs upstream and downstream of *XC_0531* are *XC_0529* (encoding an acetyl-CoA carboxylase biotin carboxyl carrier protein subunit), *XC_0530* (encoding a 3-dehydroquinate dehydratase) and *XC_0532* (encoding a divalent cation tolerance protein) (Fig. 2a). A redox active Cys-X-X-Cys motif, the conserved motif for the Dsb protein family [36], is embedded between the 686th and 683th amino acid residues of the *XC_0531* protein (Fig. 2b). Transmembrane helices analysis of *XC_0531* showed that the protein spans the membrane nine times with its N terminus facing the cytoplasm and C terminus facing the outside (Fig. 2c), which is in accordance with the identified Dsb proteins [37]. These results suggest that the deduced protein of the ORF *XC_0531* may be a DsbD protein. Another Dsb family protein encoded by *XC_3579* in *Xcc* was used in this study to compare the function of *XC_0531*. *XC_3579* is predicted to encode protein DsbC, a disulfide isomerase. It displays a 42% identity and 57% similarity to the thiol:disulfide interchange protein DsbC of *Shewanella oneidensis* MR-1 (NCBI-Protein ID: NP_716580, encoded by the *SO_0951* gene), and 39% identity and 59% similarity to the DsbC protein (NCBI-Protein ID: NP_252426) of *Pseudomonas aeruginosa* PAO1.

### Mutant of *XC_0531* is deficient in biosynthesis of c-type cytochromes

**Fig. 3:**
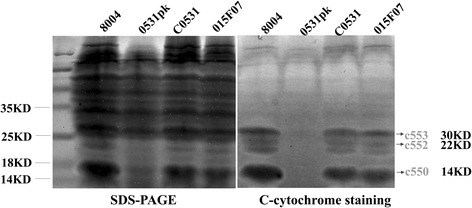
Heme staining of soluble membrane-bound proteins. Soluble membrane-bound proteins in 8004, 0531pk, C0531 and 015F07 were analyzed by heme staining after SDS-PAGE. Equivalent amounts of protein were loaded in each lane, shown stained in panel left. The membrane components were stained (Mrs, 14 kDa, 22 kDa and 30 kDa) in panel right. Lanes contained total soluble membrane-bound heme from 8004, 0531pk, C0531 and 015F07, as indicated. The positions of molecular mass markers are indicated

*XC_0531* encodes a putative, c-type cytochrome biogenesis protein homolog to DsbD (http://xgb.leibniz-fli.de). SDS-PAGE and heme staining were used to confirm this molecular property. Heme staining showed four major bands in the wild type (8004), the complemented strain (C0531) and deletion of *XC_3579* (015F07), but no band was observed in the *XC_0531* mutant (0531pk) (Fig. 3). Three of the four bands had similar molecular weights of c-type cytochromes, including 30 kD (might be cytochrome c553), 22 kD (might be cytochrome c552) and 14 kD (might be cytochrome c550) [38].

**Fig. 4:**
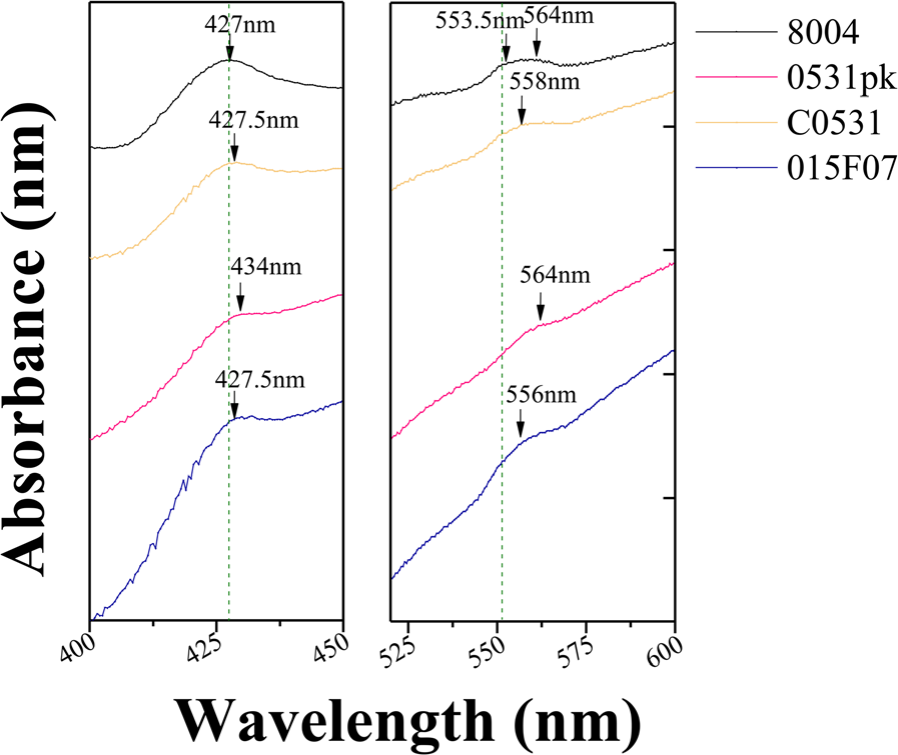
Room-temperature absorption spectra of soluble cytochromes in the membranes of 8004, 0531pk, C0531 and 015F07. The c-type cytochromes showed peaks at about 552 nm and the b-type cytochromes at about 560 nm. Samples were reduced with sodium dithionite or oxidized with ammonium persulfate. The spectra were reduced-minus-oxidized spectra at equal protein concentrations. Absorption maxima were indicated by arrows

Redox difference spectroscopy was used to further analyze the nature of the *Xcc* cytochromes. A peak absorption at ~427 nm was observed in the wild type, C0531 and 015F07, but in the mutant (0531pk) the peak was shifted to 434 nm (Fig. 4). Characteristic of the porphyrin compounds Soret band is the peak absorption at ~420 nm [39]. In addition, the wild type strain (8004) showed a peak plateau at absorption from 553.5 nm to 564 nm, while the mutant (0531pk) had only one peak at the absorption of 564 nm, which is typical of b-type cytochromes [40] (Fig. 4). A previous study has shown that cytochromes c and b have two peaks between 550 nm and 560 nm, when absorption was measured at ultralow temperature [40]. It is likely that the plateau we observed in wild type is composed of two peaks of cytochromes c and b, but the peaks were not resolved at the room temperature we used for the measurement.The complement strain (C0531) and 015F07 had a wide peak similar to wild type with the maximum absorption at 558 nm and 556 nm, respectively. These results further suggest that *XC_0531* not *XC_3579* encodes a protein related to the biosynthesis of c-type cytochromes.

### *XC_0531* affects the disulfide bond formation and copper tolerance

**Fig. 5:**
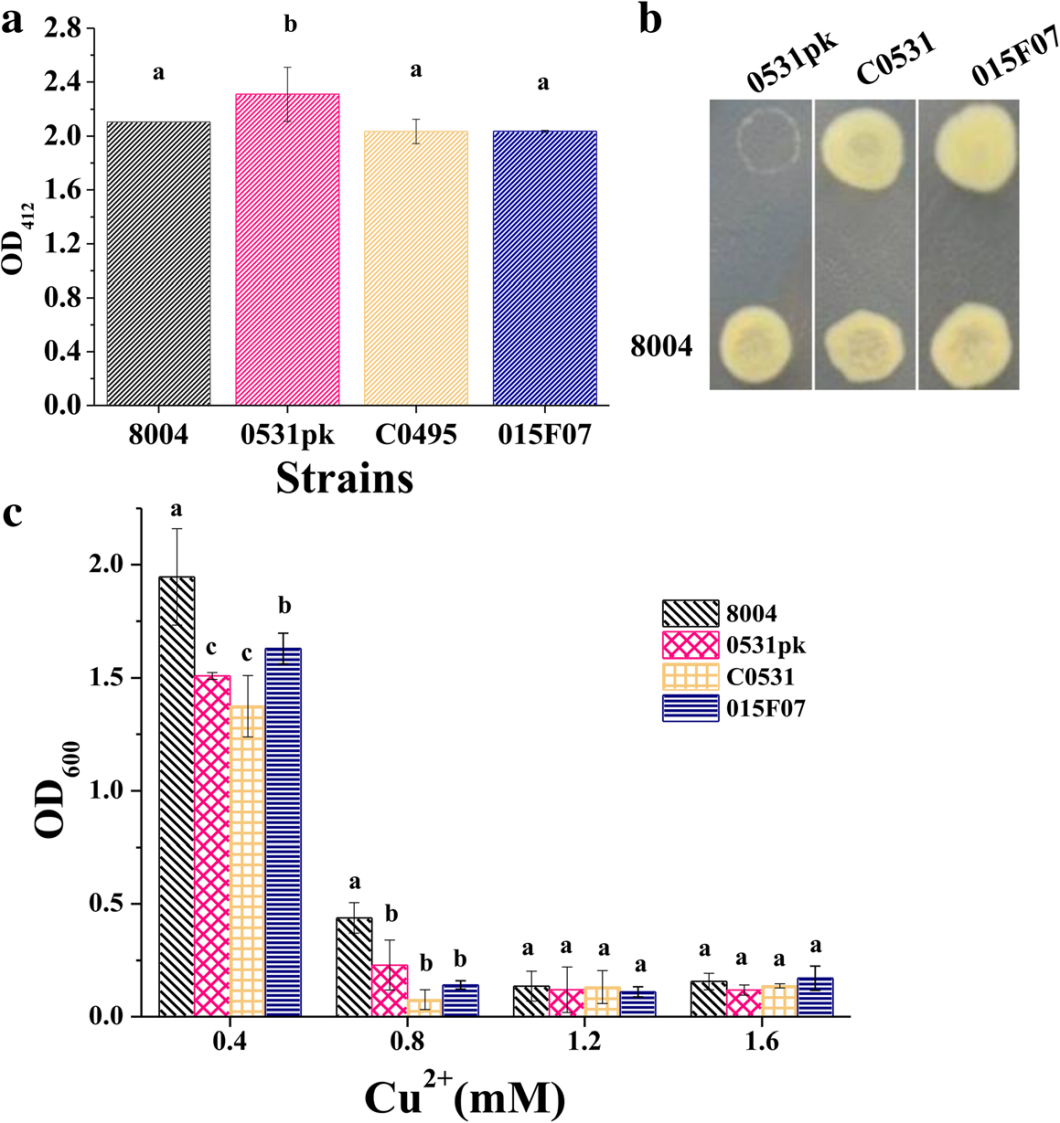
Analysis of the periplasmic disulfide oxidoreductase activity and copper tolerance of *Xanthomonas campestris* pv. *campestris* strains. **a** Accumulation of proteins with reduced cysteines determined by the Ellman’s reaction analysis. Value is the mean ± standard deviation from three repeats; OD_412_ = optical density at 412 nm. **b** Dithiothreitol (DTT) sensitivity detection. Overnight culture (2 μl) of each *X. campestris* pv. *campestris* strain was spotted onto NYGA supplemented with DTT to a final concentration of 4 mM and incubated at 28 °C. Photographs were taken 2 days after inoculation. Three plates were inoculated in each experiment and each experiment was repeated three times. Data presented were from representative plates and similar results were obtained in all plates of an experiment or in the plates of other independent experiments. **c** Copper tolerance of wild-type *Xcc* 8004, 0531pk, C0531 and 015F07. Overnight cultures were cultured in NYG containing various concentrations of CuSO_4_ for 24 to 48 h at 30 °C. OD_600_ were test. The values are averages of three independent experiments. Different letters indicate significant differences at a level of *α* < 0.05 based on Duncan’s test by one way-ANOVA

To determine whether disruption of the *XC_0531* gene has any effect on the formation of disulfide bonds in the periplasmic proteins, we examined the free thiol groups in the periplasm by measuring the accumulation of proteins with reduced cysteines in periplasm using Ellman’s reaction method [41]. The result showed that the 0531pk had an increased accumulation of proteins with free thiols (Fig. 5a). In addition, the mutant strain 0531pk was more sensitive to the strong reducing agent DTT than the wild type strain (Fig. 5b). These phenotypes of the mutant were restored to wild type in the complement strain. The mutant 015F07 showed an accumulation similar to the wild type. These results suggest that ORF *XC_0531* affects the disulfide bond formation, similar to Dsb proteins that catalyze disulfide bond formation in the periplasm.

In *Escherichia coli*, DsbD protein functions in both cytochromes c and in copper tolerance [12]. The analysis of copper tolerance of *Xcc* showed after 2 days’ culture in the presence of copper (0.4 mM or 0.8 mM of CuSO_4_), the OD_600_ of wide type is higher than strains 0531pk, C0531 and 015F07 (Fig. 5c). This indicated that 0531pk, C0531 and 015F07 were sensitive to copper_._ *XC_0531* and *XC_3579* functioned in copper tolerance. We supposed the complement strain might grow slower in double pressures with an introduced plasmid artificially and metal ions that did not compensate the phenotype of *XC_0531* mutant in the test of Cu stress.

### Deletion of *XC_0531* delays cell growth and reduces EPS production

**Fig. 6:**
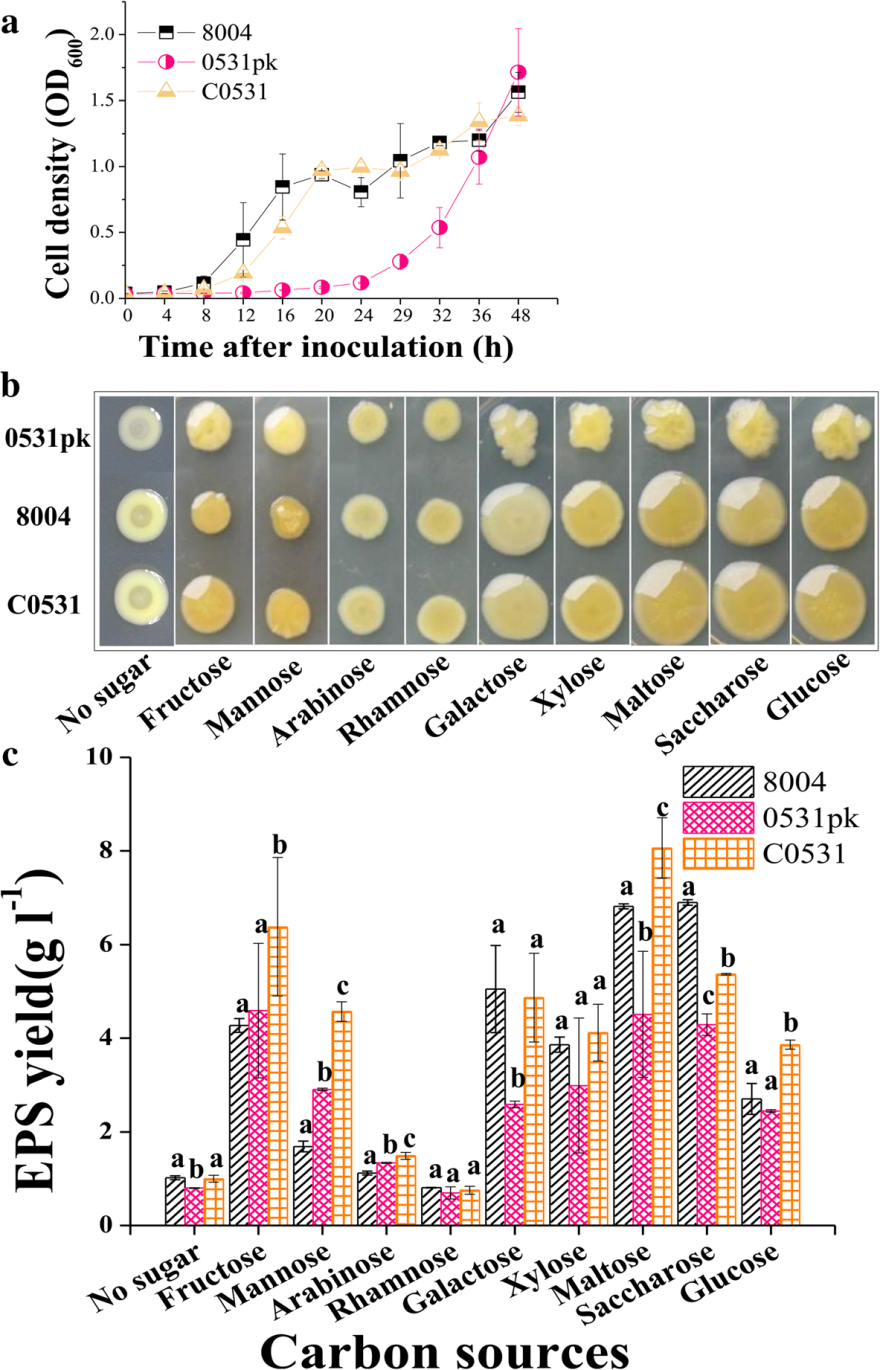
Cell growth and EPS production of *Xcc* strains at different sugars. **a** The growth of bacteria in radish leaf tissues was determined at OD_600_ in NY medium. **b** Strains were stabled into the plates followed by incubation at 28 °C for 4 days. **c** Strains in100 ml NY medium supplemented with or without 2% (wt/vol) various sugars at 28 °C with shaking at 200 rpm for 3 days. EPS was precipitated from the culture supernatant with ethanol, dried and weighed. Significant difference (*P* < 0.05) tested by one way-ANOVA. Different letters within one sugar indicate significant differences at a level of *α* < 0.05 based on Duncan’s test by one way-ANOVA

To further understand the physiological role of *XC_0531* in *Xcc*, we compared the growth rates between the wild type (8004) and the mutant (0531pk) in NYG medium. As shown in Fig. 6a, the mutant grew much slower within the first 24 h of culture. The complemented strain showed a similar growth curve as the wild type. Cell growth was further tested on NY agar plates supplied with different carbon sources, including fructose, mannose, arabinose, rhamnose, galactose, xylose, maltose, saccharose and glucose. The mutant and the wild type grew similarly in colony size on fructose, mannose, arabinose and rhamnose. However, the colonies of mutants were smaller than the wild-type strain when growing on plates with galactose, xylose, maltose, saccharose and glucose (Fig. 6b). The complemented strain formed colonies similar to those of the wild-type on plates. These results suggest that the *XC_0531* mutant is deficient in EPS production, which is critical to colony growth. To confirm that the *XC_0531* is involved in EPS production, strains were cultured in NY liquid medium supplemented with 2% various sugars for 3 days. As summarized in Fig. 6c, the EPS production in the mutant was significantly lower than the wild type (*P* < 0.05) with galactose, xylose, maltose and saccharose. In addition, the EPS yield of the complemented strain showed no significant difference (*P* > 0.05) from that of the wild-type when cultured in medium containing these five carbohydrates. These results further confirm that the *XC_0531* is involved in EPS production in *Xcc*. We also compared the colonies of Tn5 insertions in gene *XC* ***_*** *3579* encoding DsbC (015F07, 045F12 and 142E11) to the wild type in the plates containing glucose. There was no significant difference between mutants and wild type. The EPS level in *XC_3579* Tn5 gus mutants also showed similar characteristics to wild type (Additional file 2: Figure S1).

### Mutant of *XC_0531* is deficient in mobility

**Fig. 7:**
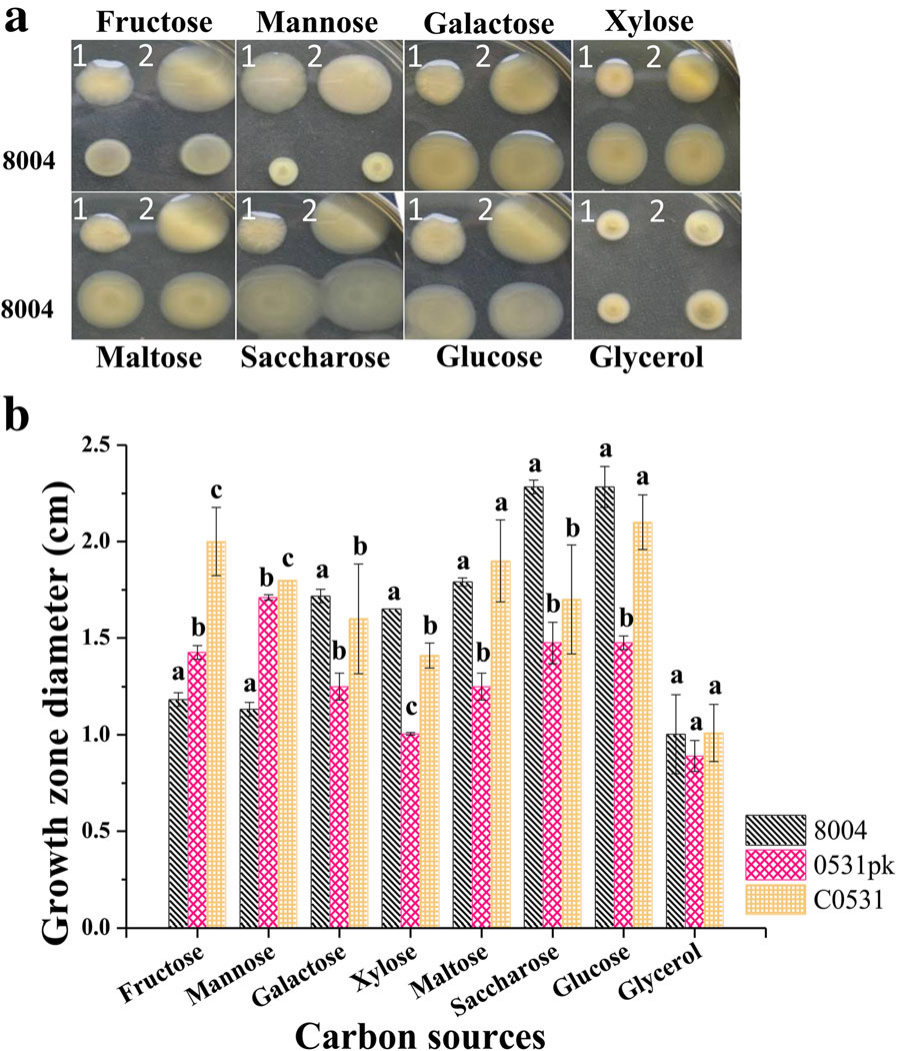
Test of the cell motility. Three μl of overnight culture (OD_600_ of 1.0) of each *Xcc* strain was inoculated onto NY plates containing 2% different sugars and 0.6% agar, and then incubated at 28 °C for 4 days to detect swarming motility. **a** Growth zone of each strain and **b** Quantitative measurement of growth zone in **a**. 1, 0531pk; 2, C0531. The experiment was repeated at least three times. Different letters within one sugar indicate significant differences at a level of *α* < 0.05 based on Duncan’s test by one way-ANOVA

The cell motility of the *XC_0531* mutant was also significantly reduced compared to the wild type using different sugars (*P* < 0.05) such as galactose, xylose, maltose, saccharose and glucose. The growth zone diameter of mutant 0531pk is 1.48 ± 0.04 cm with glucose as the carbon source and the wild type is 2.28 ± 0.11 cm (Fig. 7a and b). We don’t know why the mobilities of mutant and completment strains formed colonies larger than those of the wild type on plates containing fructose and mannose as sole carbon source (Fig. 7a and b). There was no difference in growth zone diameter between *XC_3579* Tn5 mutants’ and wild type (Additional file 3: Figure S2). These results suggest that the *XC_0531*, but not *XC_3579* affects the motility of *Xcc*.

### Mutant of *XC_0531* reduces extracellular enzyme activities

**Fig. 8:**
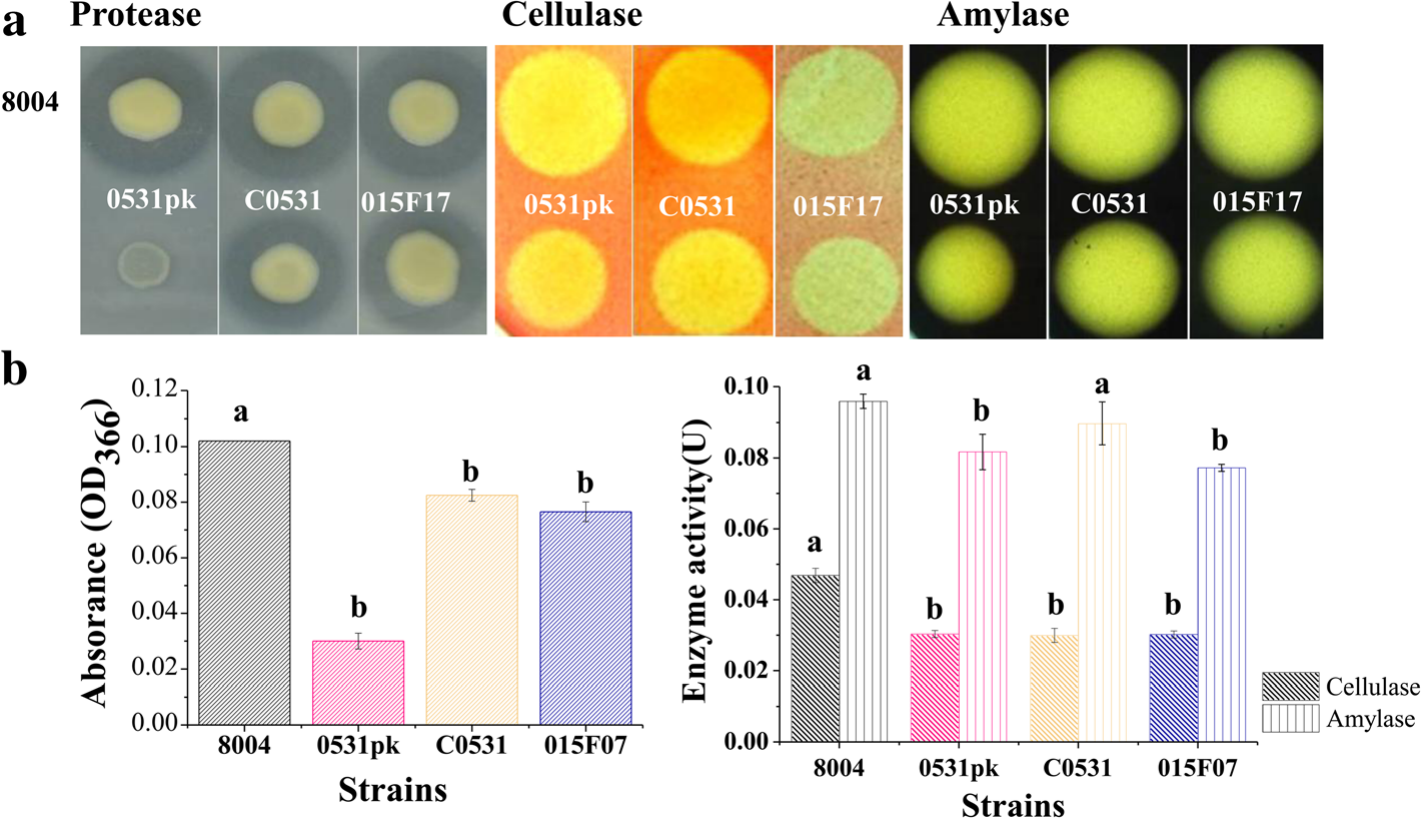
Examination of extracellular enzymes’ activities. **a** Overnight culture (3 ul) was spotted onto NYGA plates containing 1% skim milk (for protease), after incubation at 28 °C for 24 h, plates were photographed. For cellulose, 3 ul overnight culture was spotted onto NYGA plates containing 0.5% carboxymethyl cellulose after incubation at 28 °C for 48 h, plates were stained by I_2_/KI (0.08 M I_2_, 3.2 M KI) and washed by 70% ethyl alcohol. For amylse, 3 ul overnight cultures were spotted onto NYGA plates containing 0.1% starch incubation at 28 °C for 24 h. The plates were stained by 0.1% Congo Red and then washed 2 times. At last plates were destained by 1 M NaCl solution. **b** For extracellular protease activity, the method described by Swift and associates was used. 10 μl of enzyme-containing extracts were added to 200 μl of indicator buffer containing 1% (wt/vol) carboxymethyl cellulose as the substrate. The reactions were carried out for 30 min at 28 °C. The released reducing sugars were measured as D-glucose equivalents, as described by Miller. One unit (U) of the cellulase (endoglucanase) activity was defined as the amount of enzyme releasing 1 μmol of reducing sugar per minute. Amylase activity quantification was conducted in the same way as for the cellulose (endoglucanase) measurement, except that the substrate 1% (wt/vol) CMC was replaced by 1% (wt/vol) starch solution. Different letters within one enzyme activity assay indicate significant differences at a level of *α* < 0.05 based on Duncan’s test by one way-ANOVA

Spots in the milk plates showed the transparent circle formed by mutant 0531pk is smaller than the wild type (Fig. 8a). The protease activity assay showed that the transparent circle in the mutant 0531pk was significantly smaller than that of the wild type (Fig. 8a and B). The complemented strain C0531 recovered the extracellular protease activity to that in the wild type in the plate assay but not in the liquid activity test (Fig. 8a and b). The Tn5 mutant 015F07 also showed a smaller transparent circle (Fig. 8a and b). This suggests that both *XC_0531* and *XC_3579* are involved in extracellular protease activity of *Xcc*.

In the test of extracellular cellulose and amylase activities, smaller transparent circles of 0531pk and 015F07 were compared to wild type 8004. The Tn5 mutant 015F07 showed a smaller transparent circle and lower protease activity compared to wild type) (*P* < 0.05) (Fig. 8a and c). The complemented strain C0531, however, did not fully recover the extracellular cellulase activity to the wild type (Fig. 8c).

### The relationship between *dsbD* and *dsbA*, *dsbB*, or *dsbC*

To verify whether the function of gene *dsbD* in pathogenicity of *Xcc* is due to affect genes *dsbA* (*XC_0674* and *XC_0675*), *dsbB* (*XC_3314*) or *dsbC* (*XC_3579*) expression, we checked transcript levels of *dsbA*, *dsbB* and *dsbC* in *dsbD* deleted and complemented strains. Real-time quantitative PCR was employed to assay the *dsbA*, *dsbB* and *dsbC* transcripts in *dsbD* deleted and complemented strains grown in NYG medium. The results showed that the transcription levels of *dsbA*, *dsbB* and *dsbC* between these two strains differed only within 1.1 folds (*P* > 0.05) (Additional file 4: Figure S3), suggesting that the *dsbD* expression of *Xcc* does not affect other *dsb* genes.

## Discussions

Iron ions in the heme act as an activator to catalyze H_2_O_2_ to produce O_2_ and then the O_2_ oxidize TMBZ to turn the heme into blue [42]. Sequence analysis suggests that *XC_0531* likely encodes protein DsbD. *dsbD* deletion caused deficient in c-type cytochromes, similar to a report in *Brassica japonicum* cox3, lacking all soluble c-type cytochromes [43]. By complement pL0531, all the deficit phenomena could be reversed to those phenomena in wild type. In the *Rhodobacter species* protein DsbD provides electrons to apoCyt C [13]. DsbD is also reported in *Neisseria meningitidis* to provide reducing power to DsbC that shuffles incorrect disulfide bond as well as to the periplasmic enzymes that reduce apo-cytochrome c (CcsX) [44]. In our results, no soluble heme was observed in the *XC_0531* mutant, suggesting that *XC_0531* deletion might affect the process of electrons to apoCyt C and then the process of heme ligation to the apoCyts C and the maturation of cytochromes C will be disturbed. Further test of cytochromes C using reduced-minus-oxidized spectra showed the characteristics of the porphyrin compounds Soret band in *dsbD* mutant red shift to 434 nm compared to that in wild type. The phenomena of red shift usually appeared due to the chromophores with unsaturated group such as heme with unsaturated group. The Soret band is a characteristic of porphyrin, therefore, the red shift of Soret band suggests that the proximal ligands with stronger ability of supply electrons (more electron clouds) appeared in the sole compand study [45]. Each strain could be supposed to a compand and the gene deletion could be regard as a replace of proximal ligands. The *dsbD* deletion could form a larger electrons cloud that could not be transferred smoothly. This also implied the process of electrons to apoCyt C was interrupted.

The c-type cytochromes are needed in the cellular respiratory chain for the electrons transfer to provide energy to the cells. Deletion of *dsbD* might affect the respiration of *Xcc* by losing synthesis of c-type cytochromes. This is one of the reasons that the *dsbD* deletion strain of *Xcc* grows slower, with poor carbohydrate’ utilization (such as galactose, xylose, maltose, saccharose and glucose), decreased enzymes activity (such asprotease, cellulose and amylase) and less production of EPS (under different sugar, such as, galactose, xylose, maltose, saccharose and glucose). EPS is the important pathogenic factor to the host plant. So the *XC_0531* deletion also weakened virulence to plants. On the other hand, Dsb proteins of gram-negative bacteria are a group of proteins that catalyze disulfide bond formation in the periplasm [17]. DsbD has the capacity to reduce disulfide bonds and keeps the DsbC in a reduced state [46, 47]. Virulence factors such as extracellular enzymes and EPS are secreted via type II secretion system (T2SS) in gram-negative bacteria [48, 49]. The proteins of T2SS such as XpsD, XpsM, and XcsN located in periplasm all have cysteine residues and their assembly and cysteine residues modifications were completed in periplasm [35, 48–51]. Disulfide bonds are important for maintaining the structure of proteins. *dsbD* deletion might decrease the secretion and activities of extracelluar enzymes by affecting synthesis of the contents of T2SS and extracellular enzymes. These all affected the pathogenicity to plants.

Proteins DsbC and DsbA could affect extracellular enzyme formation in *Pseudomonas aeruginosa* or *Erwinia carotovora* [52–54]. In our results, we found the extracellular enzyme activities such as protease, cellulose and amylase all decreased in mutant of *dsbD* but not *dsbC*. This indicated that the decrease of extracellular enzyme activities in *dsbD* mutant is not related to DsbC. DsbD protein is also reported in *Neisseria meningitidis* to provide reducing power to the periplasmic enzymes relative to reduction of apo-cytochrome c [44]. Cytochrome c is a part of respiratory chain. Respiratory chain is required to maintain oxidized states of the DsbA-DsbB disulfide bond formation system in aerobically growing *Escherichia coli* cells [55]. Our qPCR results also showed that in complemented strain C0531 transcription levels of *dsbA*, *dsbB* and *dsbC* were the same as that in mutant 0531pk (Additional file 4: Figure S3). Gene *dsbD* expression in C0531 did not affect transcription of *dsbA*, *dsbB* or *dsbC.* This further demonstrated *dsbD* deletion might affect the protein modification process of DsbA-DsbB disulfide bond formation system by deficiency of cytochromes C on the protein level not the mRNA level.

Our results suggest that, similar to DsbB [17], DsbD affects the formation of disulfide bond. Proteins DsbD and DsbC functioned in copper tolerance like those in *Escherichia coli* [12]. The complement strain did not compensate the phenotype of *XC_0531* mutant. We believe that the complement strain might grow slower in double pressures with an introduced plasmid and the presence of copper stress. Deletion of *XC_3579* (encodes a DsbC protein) gave no significant differences to the wild type on the EPS production, utilization of carbohydrates, virulence to plant and c-type cytochromes biogenesis. *dsbC* encoded disulfide isomerase could correct misfolded proteins [12]. Its possible recessive nature (functions only in the presence of misfolded proteins) may explain why its deletion did not affect any phenomena in *Xcc*.

## Conclusions

The phytopathogenic *Xanthomonas campestris* pv. *campestris* is a gram-negative bacterium and the causal agent of black-rot disease of cruciferous crops. This study is of important to *Xanthomonas pathogenesis*. We confirmed the function of gene *XC_0531*, which encodes a DsbD like protein, a protein correlated with c-type cytochrome biogenesis, polysaccharide production and the virulence to plants. It is also probably related to disulfide bonds modification of proteins in type II secretion system (T2SS). These results will provide data to better understand the molecular mechanism of pathogenesis related to *Xanthomonas campestris* pv. *campestris*.

## Additional files

Additional file 1: Table S1. Primers used in this study. (TIFF 3416 kb)

Additional file 2: Figure S1. EPS production of *Xcc* strains at glucose plate. Strains were stabled into the plates followed by incubation at 28 °C for 4 days. Strains in 100 ml NY medium supplemented with 2% (wt/vol) various sugars at 28 °C with shaking at 200 rpm for 3 days. EPS was precipitated from the culture supernatant with ethanol, dried and weighed. Different letters within one sugar indicate significant differences at a level of *α* < 0.05 based on Duncan’s test by one way-ANOVA. (TIFF 5151 kb)

Additional file 3: Figure S2. Test of the cell motility. An overnight culture (OD_600_ of 1.0) of each *Xcc* strain was inoculatedonto NY plates containing 2% glucose and 0.6% agar using a toothpick, and then incubated at 28 °C for 4 days to detect swarming motility.1, 015F07; 2, 045F12; 3, 142E11. (TIFF 2153 kb)

Additional file 4: Figure S3. Real-time quantitative PCR analysis. Real-time quantitative PCR to analyze the expression of the genes *dsbA* (*XC_0674* and *XC_0675*), *B* (*XC_3314*) and *C* (*XC_3579*) in strains 0531pk and C0531. RNA was isolated from cultures of *Xcc* strain 0531pk and C0531 grown in NYG medium alone for 24 h. The relative mRNA levels of *dsbA* (*XC_0674* and *XC_0675*), *B* (*XC_3314*) and *C* (*XC_3579*) in C0531 was calculated with levels of the corresponding transcription in cells of 0531pk (values was specified as 1). Values given are the means ± SD of triplicate measurements. (DOCX 13 kb)

## Abbreviations

ANOVA: One-way analysis of variance
DTNB: 5, 5′-dithiobis-[2-nitrobenzoic acid]
DTT: dithiothreitol
EPS: extracellular polysaccharide
Kan: kanamycin
NCBI: National Center for Biotechnology Information
Rif: rifampicin
Tet: tetracycline
TMBZ: 3,3′,5,5′- tetramethyl benzidine
*Xcc*: *Xanthomonas campestris* pv. *campestris*
