# Supplementary figures and images for: XC_0531 encodes a c-type cytochrome biogenesis protein and is required for pathogenesis in Xanthomonas campestris pv. campestris
Source: BMC Microbiol. 2017 Jun 27;17:142. doi: 10.1186/s12866-017-1056-9 (PMC5488342; doi:10.1186/s12866-017-1056-9)

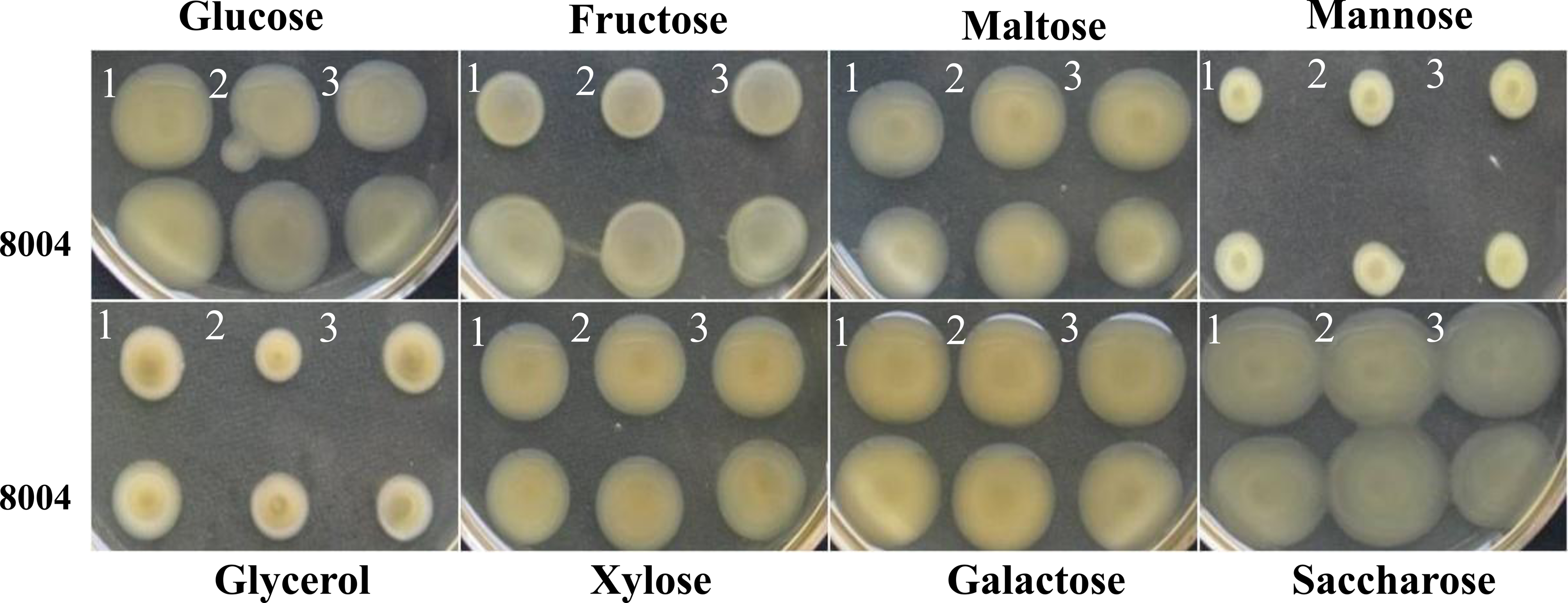

Supplement: Supplementary file 1 — Primers used in this study. (TIFF 3416 kb) [file 12866_2017_1056_MOESM1_ESM.tif]

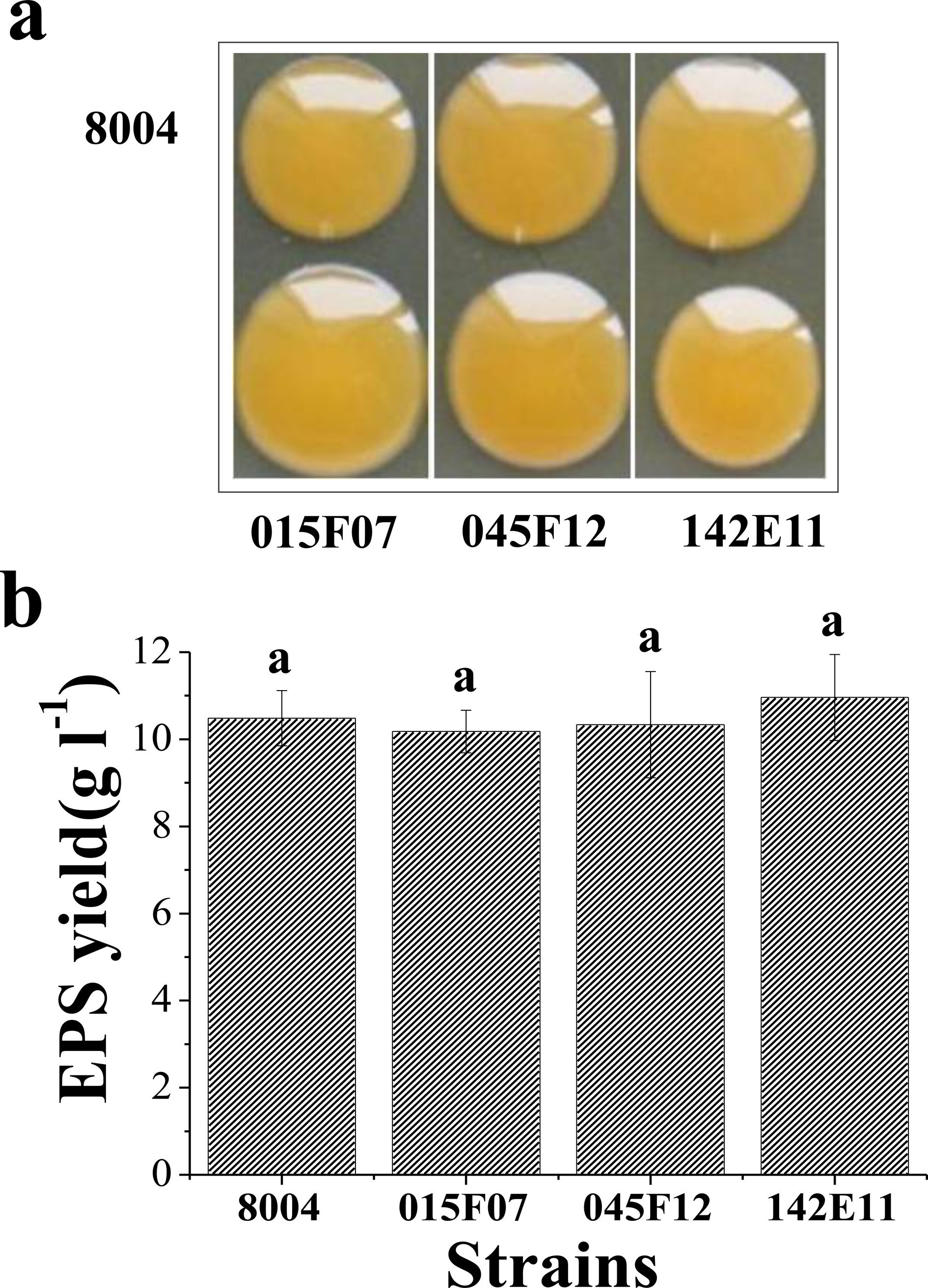

Supplement: Supplementary file 2 — EPS production of Xcc strains at glucose plate. Strains were stabled into the plates followed by incubation at 28 °C for 4 days. Strains in 100 ml NY medium supplemented with 2% (wt/vol) various sugars at 28 °C with shaking at 200 rpm for 3 days. EPS was precipitated from the culture supernatant with ethanol, dried and weighed. Different letters within one sugar indicate significant differences at a level of α < 0.05 based on Duncan’s test by one way-ANOVA. (TIFF 5151 kb) [file 12866_2017_1056_MOESM2_ESM.tif]

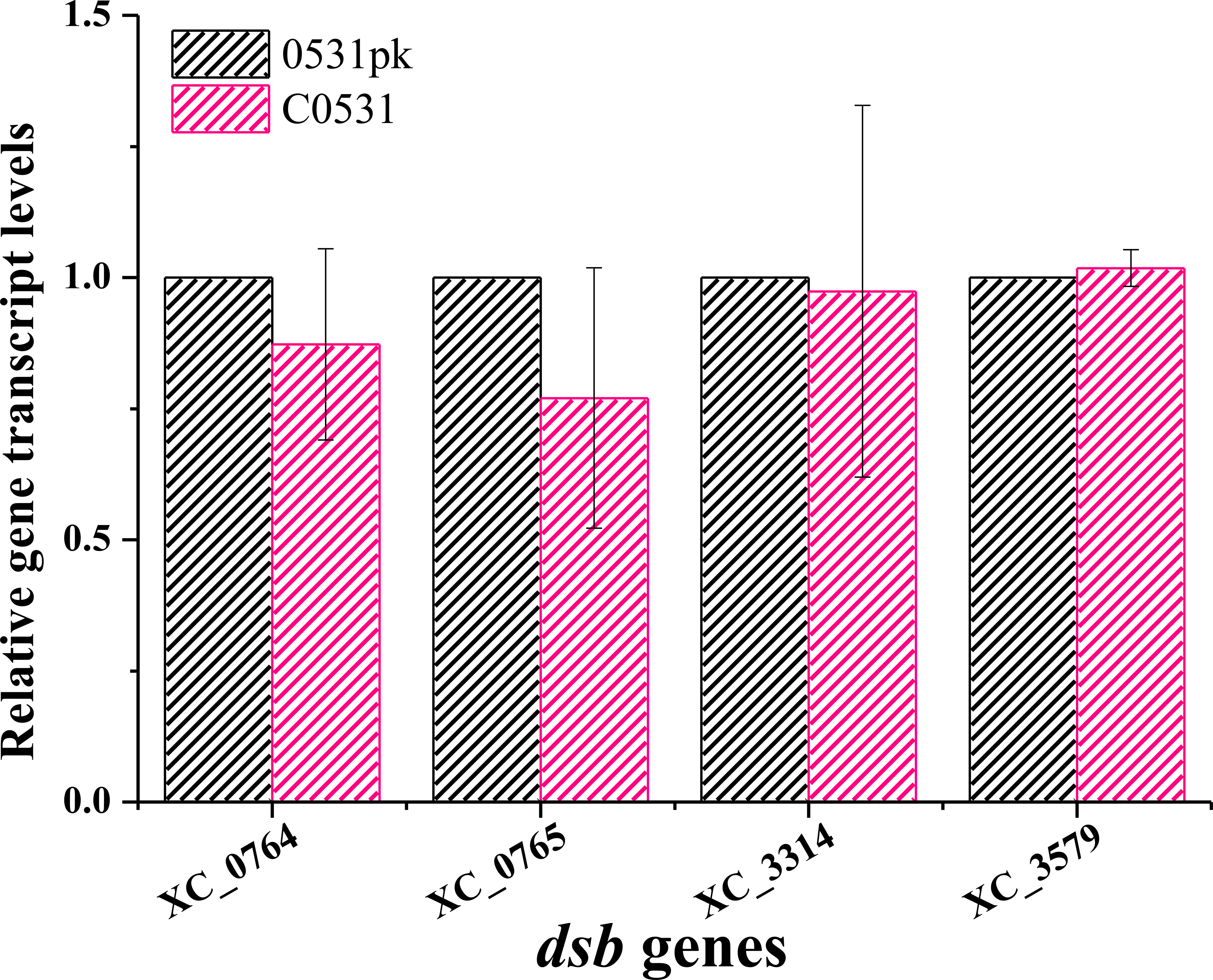

Supplement: Supplementary file 3 — Test of the cell motility. An overnight culture (OD600 of 1.0) of each Xcc strain was inoculatedonto NY plates containing 2% glucose and 0.6% agar using a toothpick, and then incubated at 28 °C for 4 days to detect swarming motility.1, 015F07; 2, 045F12; 3, 142E11. (TIFF 2153 kb) [file 12866_2017_1056_MOESM3_ESM.tif]
