# Supplementary material for: XC_0531 encodes a c-type cytochrome biogenesis protein and is required for pathogenesis in Xanthomonas campestris pv. campestris
Source: BMC Microbiol. 2017 Jun 27;17:142. doi: 10.1186/s12866-017-1056-9 (PMC5488342; doi:10.1186/s12866-017-1056-9)
Supplement: Supplementary file 4 — Real-time quantitative PCR analysis. Real-time quantitative PCR to analyze the expression of the genes dsbA (XC_0674 and XC_0675), B (XC_3314) and C (XC_3579) in strains 0531pk and C0531. RNA was isolated from cultures of Xcc strain 0531pk and C0531 grown in NYG medium alone for 24 h. The relative mRNA levels of dsbA (XC_0674 and XC_0675), B (XC_3314) and C (XC_3579) in C0531 was calculated with levels of the corresponding transcription in cells of 0531pk (values was specified as 1). Values given are the means ± SD of triplicate measurements. (DOCX 13 kb) [file 12866_2017_1056_MOESM4_ESM.docx]

**Table S1 Primers used in this study**

| **Primers** | **sequences（5’→ 3’）** | Description |
| --- | --- | --- |
| Tn5-F | CGGGAAACGTCTTGCTCGAGGCC | Verification of Tn5-inserted mutant |
| Tn5-R | TGCTTTGCCACGGAACGGTCTGC |  |
| C0531-F | AAGCTT GCGGCAGTCAGTACTGAT | Construction of Plasmid pLC0531 |
| C0531-R | GGATCC TCCCATGCTGAATCAAGC |  |
| 0531-F | GGATCC AGCACGCCGGTAAAGAAG | Deletion of *XC_0531* |
| 0531-R | GGATCC GATCCTGTCGCTGAAGGT |  |
| XC3580F | AGCAGTCCCGCCGAACGCCG | Verification of Tn5-inserted mutant *XC_3579* |
| XC3578R | CAGCAACAATCCACCCTCGC |  |
| P18conF | GCCGATTCATTAATGCAGCTGGCAC | Verification of construction |
| XC0764F | GGCACCGATTACCTGGATAT | qPCR analysis of *XC_0764* |
| XC0764R | AGACGAAATGCACATCCGAC |  |
| XC0765F | CCTGTTTTCCCGTCTGTCGC | qPCR analysis of *XC_0765* |
| XC0765R | AACACTTCCGCCACTTCGAC |  |
| XC3314F | CATTCCGTTGGGGTTTTCGG | qPCR analysis of *XC_3314* |
| XC3314R | AGCAGGAACAGCAAGGCCAA |  |
| XC3579F | TATCGTCTTGCCATCGCCGC | qPCR analysis of *XC_3579* |
| XC3579R | CGGCTTGAAATCCGGGTCCA |  |
